# Gut microbiota and short-chain fatty acids in pregnant women with group B *Streptococcus* colonization and their impact on neonatal outcomes

**DOI:** 10.3389/fcimb.2026.1912234

**Published:** 2026-09-10

**Authors:** Yao Zhu, Yan Ni, Yayin Lin, Xinsi Chen, Jidong Lai, Xinzhu Lin, Chao Chen

**Affiliations:** 1 Department of Neonatology, Children’s Hospital of Fudan University, Shanghai, China; 2 Key Laboratory of Neonatal Diseases, National Health Commission, Shanghai, China; 3 Department of Neonatology, Women and Children’s Hospital, School of Medicine, Xiamen University, Xiamen, Fujian, China; 4 Department of Obstetrics, Women and Children’s Hospital, School of Medicine, Xiamen University, Xiamen, Fujian, China

**Keywords:** group B Streptococcus, intestinal flora, late pregnancy, neonate, short-chain fatty acids, umbilical cord blood IL-6

## Abstract

**Background:**

Group B *Streptococcus* (GBS) frequently colonizes the gastrointestinal and lower reproductive tracts in pregnant women. However, research has predominantly addressed its influence on the vaginal microbiota, leaving its effects on the gut ecosystem and the consequent implications for neonatal outcomes largely unexplored.

**Methods:**

In this prospective study, 87 pregnant women were enrolled at the Women and Children’s Hospital of Xiamen University from March 2022 to October 2023 and divided into GBS-positive and GBS-negative cohorts according to their colonization status. Fecal samples were collected to complete 16S rRNA gene sequencing and fecal Short-chain fatty acids (SCFAs) content determination. The pregnancy outcomes and clinical prognosis information of the newborns were followed up. Intestinal microbiota composition and SCFAs levels were compared between GBS-positive and GBS-negative pregnant women, and their respective correlations with pregnancy and neonatal outcomes were evaluated.

**Results:**

Significant differences in the genera *Ruminococcus*, *Klebsiella*, and *Intestinibacter* were observed between GBS-positive and GBS-negative group. SCFAs-targeted metabolomic analysis revealed that fecal butyrate concentration were significantly lower in GBS-positive group than in GBS-negative group. Association analysis revealed a significant positive correlation between intestinal butyrate concentration and the abundance of *Ruminococcus* (*ρ* = 0.216, *P* = 0.045). Spearman’s correlation analysis showed that *Ruminococcus* abundance and butyrate concentration were positively correlated with birth weight percentile-Fenton of newborn (*ρ* = 0.390, *P* < 0.001; *ρ* = 0.403, *P* < 0.001) and negatively correlated with umbilical cord blood IL-6 level (*ρ* = -0.229, *P* = 0.033; *ρ* = -0.445, *P* < 0.001). After adjusting for confounding factors including GBS colonization, regression analysis showed that increased butyrate concentration was an independent protective factor against elevated IL-6 (a*β*: -27.823, 95%*CI*: -50.041~-5.606), whereas increased incidence of chorioamnionitis was an independent risk factor for elevated IL-6 (a*β*: 30.155, 95%*CI*: 2.556~57.754).

**Conclusions:**

GBS colonization in late pregnancy is associated with disruptions in the gut microbiota and its metabolic profiles, particularly with lower *Ruminococcus* abundance and diminished butyrate metabolism. Butyrate may lower neonatal inflammation through anti-inflammatory mechanisms. Therefore, targeting *Ruminococcus* or the butyrate metabolic pathway should be viewed as a hypothesis-generating strategy to improve perinatal outcomes in GBS-colonized pregnant women.

## 1 Introduction

Group B *Streptococcus* (GBS) is a significant pathogen in perinatal infections, contributing to adverse pregnancy outcomes and neonatal invasive infections (Gonçalves et al., 2022). Currently, universal late-pregnancy GBS screening and intrapartum antibiotic prophylaxis (IAP) have been widely implemented, substantially reducing the incidence of early-onset GBS infection in newborns (ACOG, 2020; Chinese Society of Perinatal Medicine, 2021). Nonetheless, this strategy does not entirely eradicate adverse pregnancy outcomes and is linked to complications such as gut microbiota dysbiosis and antibiotic resistance (Teuscher et al., 2025; Seedat et al., 2017).

GBS commonly colonizes the gastrointestinal and lower genital tracts of asymptomatic women, with up to 30% of healthy individuals harboring this commensal bacterium in the lower digestive tract (ACOG, 2020). Given that perinatal infections are often transmitted during vaginal delivery, most studies have focused on the relationship between GBS and the vaginal microbiota. A vaginal swab culture study involving 4,025 pregnant women revealed that GBS colonization during the second trimester was associated with lower levels of Lactobacillus, Prevotella, and Candida, and higher levels of Staphylococcus (Kubota et al., 2002). Beyond the vaginal microbiota, data on the association between GBS and the host gut microbiota are limited, with most research concentrating on whether intrapartum IAP interferes with normal mother-to-infant microbial transmission. For example, one study demonstrated that newborns of GBS-positive mothers who received intrapartum IAP had a significantly lower abundance of *Bifidobacterium* in their fecal microbiota (Teuscher et al., 2025). The present study aims to compare gut microbiota differences between GBS-positive and GBS-negative pregnant women, with a focus on the relationship and functional role of gut microbiota and their metabolites in GBS colonization. Short-chain fatty acids (SCFAs), as the primary metabolites of the gut microbiota (Mukhopadhya and Louis, 2025), may suppress inflammatory responses and reduce pathogenic infections (van der and Wells, 2021). Therefore, this study primarily targets changes in SCFAs among metabolic profiles.

Accumulating evidence suggests that the maternal microbiota and its metabolites play critical roles in pregnancy maintenance, fetal immune programming, and neonatal health (Barrientos et al., 2026). Nevertheless, the relationship between maternal GBS colonization and the gut microbial–metabolic landscape, along with its possible implications for neonatal prognosis, remains poorly understood. To test this, we prospectively enrolled pregnant women with positive or negative GBS screening results during the third trimester. Our primary exposure was maternal GBS colonization status. The primary microbial outcome was the differential abundance of specific genera, and the primary metabolite outcome was the fecal concentration of SCFAs. The primary neonatal outcome was cord blood interleukin-6 (IL-6) level, a sensitive marker of fetal systemic inflammation, while birth weight percentile-Fenton (Fenton et al., 2025) was examined as a secondary clinical outcome.

Using 16S rRNA gene sequencing combined with SCFA-targeted metabolomics, we systematically analyzed alterations in the gut microbiota and SCFAs associated with GBS colonization status. We further evaluated the correlations between these microbial and metabolic changes and neonatal outcomes, aiming to elucidate whether GBS colonization influences the maternal-fetal environment via the gut microbiota–metabolite axis. This hypothesis-driven design allowed us to examine these associations in a confirmatory framework, while also generating exploratory hypotheses for future mechanistic investigations into the perinatal implications of maternal GBS colonization.

## 2 Materials and methods

### 2.1 Study design and participants

**Figure 1:**
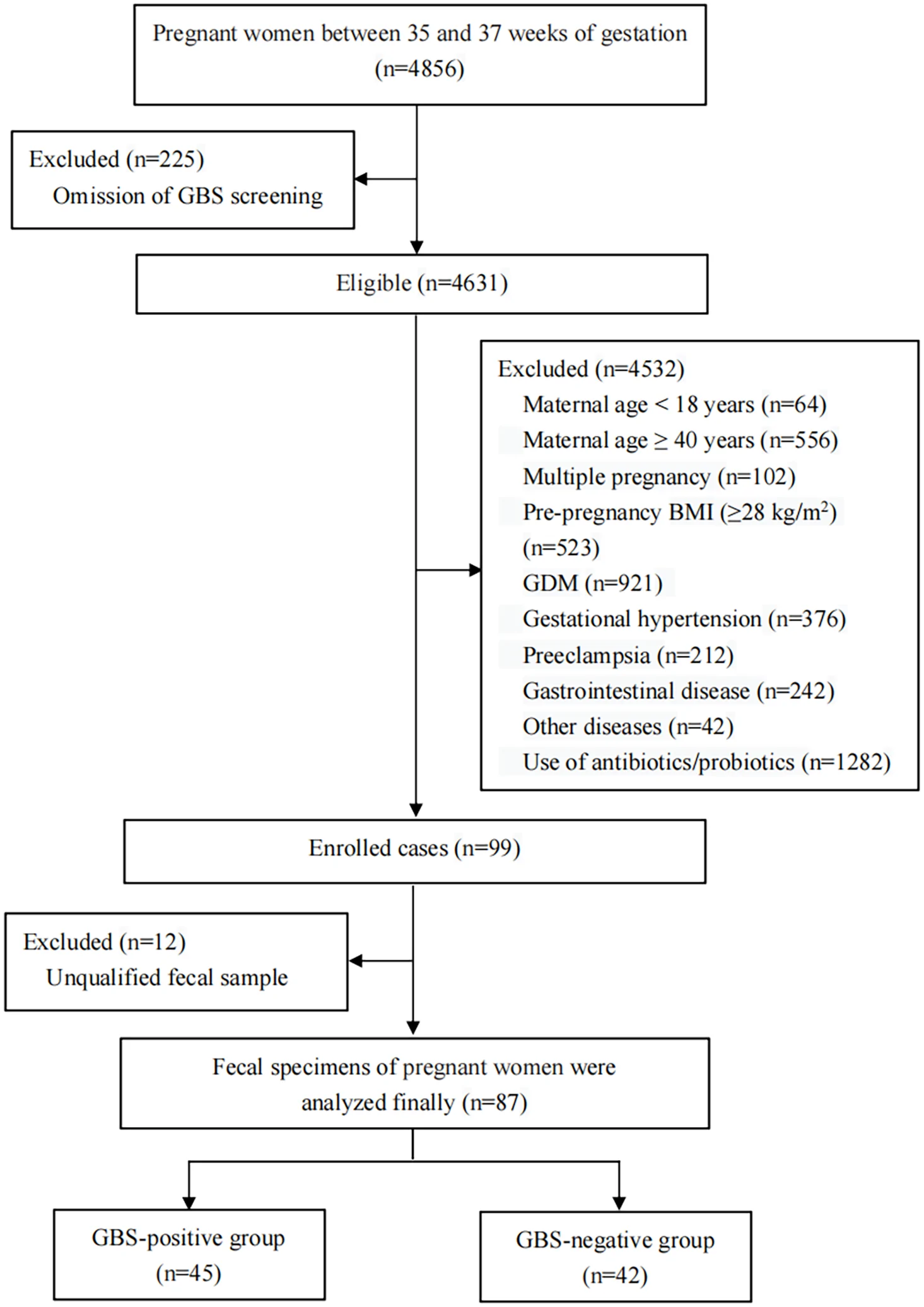
Study flow diagram.

A total of 4,856 pregnant women at 35~37 weeks of gestation were initially assessed. After excluding 225 cases who lacked GBS screening results, 4,631 cases were considered eligible. Subsequently, 4,532 cases were excluded based on the following criteria. Ultimately, 99 pregnant women were included in the final analysis (**Figure 1**). This study prospectively enrolled 45 GBS-positive and 42 GBS-negative pregnant women who received prenatal care and delivered at the Women and Children’s Hospital, School of Medicine, Xiamen University between March 2022 and October 2023. GBS-positive status was defined as a positive vaginal–rectal swab culture for GBS during the third trimester (35~37 weeks of gestation). All participants in both GBS-positive and GBS-negative groups received identical antenatal care and intrapartum management according to the institutional obstetric protocol. Specifically, the frequency of prenatal visits, fetal monitoring procedures, and mode-of-delivery assessments did not differ between the two groups. The only systematic difference in clinical management was the administration of IAP to GBS-positive women according to standard guidelines. The study was approved by the Ethics Committee of the Women and Children’s Hospital, School of Medicine, Xiamen University (No. KY-2022-036-H01; No. KY-2023-150-H01). All participants provided written informed consent in accordance with the committee’s authorization.

#### 2.1.1 Inclusion criteria

(1) agreed to sign the informed consent form and participate in the study; (2) aged between 18 and 40 years; singleton pregnancy; (3) completed vaginal-rectal swab culture for GBS during the third trimester (35~37 weeks of gestation).

#### 2.1.2 Exclusion criteria

(1) multiple pregnancy; (2) refusal to sign the informed consent form; (3) use of antibiotics, microecological preparations, or non-steroidal drugs within 8 weeks prior to enrollment; (4) obesity or underweight, defined as a pre-pregnancy body mass index (BMI) ≤ 18.5 or ≥ 28 kg/m², with gestational weight gain assessed according to the Chinese Nutrition Society’s *Gestational Weight Monitoring and Evaluation for Chinese Women* (T/CNSS 009-2021); (5) diagnosis of gestational diabetes mellitus or pregestational diabetes mellitus; (6) gestational hypertension or preeclampsia; (7) history of gastrointestinal disease; (8) presence of other major diseases, including mental disorders, hepatic or renal dysfunction, hematological diseases, autoimmune diseases, and endocrine disorders (e.g., thyroid dysfunction, Cushing’s syndrome).

### 2.2 GBS screening and culture of pregnant women

A combined vaginal-rectal swab was obtained for GBS culture. Vaginal-rectal secretions were collected from the lower third of the vagina and 2 to 5 cm above the anal sphincter of enrolled pregnant women at 35–37 weeks of gestation by swab, which were inserted into the plastic TranSwab device (provided by Qi Xing Biotechnology Company of China). Then the plastic tube was cultured at 35 °C with 5% CO_2_ for 24~48 hours. No color change in the tube was interpreted as GBS-negative, whereas a carrot-colored result was directly interpreted as GBS-positive. The culture media from all GBS Tran Swab plastic tubes were separately subcultured onto GBS two-compartment nutrient agar plates and Columbia blood agar plates and then incubated under the same conditions as described above. A carrot-colored appearance accompanied byβ-hemolysis on the two-compartment plate was considered GBS-positive. Confirmation was performed using the PHOENIX™-100 system if β-hemolysis alone or the presence of suspicious colonies on the blood agar plate.

### 2.3 Sample collection of feces

Fecal samples for microbiome analysis and SCFAs measurement were self-collected by participants during the antenatal period, specifically between the time of GBS screening (at 35~37 weeks of gestation) and the onset of labor. All maternal fecal samples were collected before the onset of labor, before the administration of IAP without premature rupture of membranes (PROM). Before fecal collection, participants emptied their bladders to avoid urine contamination. A disposable sterile pad was placed on a single-use stool collection container. Using a sterile spoon (provided with the sampling tube), an approximately 3 g portion of fresh feces was collected from the inner part of the stool that had not been exposed to air or the sterile pad. The sample was immediately transferred into a 5 mL fecal DNA collection tube, placed on ice, and transported to the laboratory. The inner part of the fecal sample was then quickly aliquoted into sterile cryotubes, each labeled with a unique identification code. Each sample was divided into two aliquots, each containing approximately 500 mg. The aliquots were immediately stored at -80 °C until further analysis. The entire process was completed within 1~2 hours, and repeated freeze-thaw cycles were avoided.

### 2.4 Fecal 16S rRNA gene sequence analysis

Microbial community genomic DNA was extracted from fecal samples using the Stool DNA Extraction Kit (Halgen, Ltd., Zhongshan, China) according to the manufacturer’s instructions. The V3~V4 hypervariable regions of the 16S rRNA gene were amplified from the extracted DNA. Conventional PCR was then performed, and the target amplicons were purified using the Gene JET Gel Extraction Kit (Thermo Fisher Scientific, USA). Sequencing libraries were constructed using the Ion Plus Fragment Library Kit and were subsequently evaluated and sequenced on an Ion S5™ XL platform. Raw reads were quality-filtered by removing reads containing ambiguous bases (N), those with an average quality score < Q20, and those trimmed to < 200 bp. Primer sequences were trimmed using Cutadapt (v4.0). Denoising, dereplication, and merged paired-end reads were processed using the DADA2 pipeline (v1.20) within QIIME 2 (2023.5). Chimeric sequences were identified and removed using the consensus method in DADA2. Amplicon Sequence Variants (ASVs) were generated following the above denoising and chimera removal steps. Taxonomic assignment of ASVs was performed using the q2-feature-classifier plugin with the classify-sklearn naive Bayes classifier against the SILVA 138.2 reference database (99% OTU sequences, trimmed to the V3–V4 region). To correct for uneven sequencing depth, the ASV feature table was rarefied to an even depth of 26,000 reads per sample; samples with fewer reads were excluded. Low-abundance ASVs (defined as relative abundance < 0.01% of total reads or present in fewer than three samples) were filtered out before downstream analysis. Alpha diversity and beta diversity were analyzed to assess microbial diversity and community structure. Linear discriminant analysis effect size (LEfSe) was performed to identify differentially abundant taxa between the GBS-positive and GBS-negative groups. All sequencing and bioinformatic analyses were conducted by Majorbio Bio-pharm Technology Co., Ltd. (Shanghai, China).

### 2.5 Fecal SCFAs quantification

After thawing, fecal samples were mixed with 1 mL of 0.5% sodium chloride-saturated phosphoric acid solution and vortexed. Subsequently, 2 mL of diethyl ether was added, and the mixture was intermittently shaken and then centrifuged at high speed for 10 min. The supernatant was filtered through a 0.22-μm syringe filter and transferred into a chromatographic vial for analysis. SCFAs quantification was performed using a 456-GC gas chromatograph (Varian, USA). All SCFAs standards (acetic, propionic, butyric, isobutyric, valeric, isovaleric, hexanoic, and isohexanoic acids) were purchased from Dr. Ehrenstorfer GmbH (Germany). The measurement of fecal SCFA levels was carried out by Majorbio Bio-pharm Technology Co., Ltd. (Shanghai, China).

### 2.6 Neonatal umbilical cord blood IL-6 analysis

Immediately after delivery of the newborn, the umbilical cord was clamped, and 2 mL of umbilical cord blood was collected from the umbilical vein using an EDTA anticoagulant tube. The sample was allowed to stand for 30 min, then centrifuged; the supernatant was aliquoted and stored at -80 °C. IL-6 levels were subsequently measured by enzyme-linked immunosorbent assay (ELISA) (Wantei Kairui Biotechnology Co., Ltd. Xiamen, China). The assay had a detection range of 1.50~5000.00 pg/mL, with a limit of detection of 1.50 pg/mL. The intra-assay and inter-assay coefficients of variation were <10.0% and <15.0%, respectively. All samples fell within the detectable range, and no values were below the limit of detection. The IL-6 data were log-transformed prior to regression analysis to address right-skewed distribution.

### 2.7 Clinical information collection and disease definition

Clinical data were collected through standardized interviews and medical records, including maternal age, residence, ethnicity, BMI at delivery, intrapartum antibiotic use, pregnancy outcomes (e.g., mode of delivery, chorioamnionitis, PROM), and neonatal outcomes (e.g., birth weight percentile-Fenton (Fenton et al., 2025), gestational age < 37 wk, early-onset GBS disease). According to clinical practice guidelines (ACOG, 2017), chorioamnionitis is suspected when intrapartum fever (≥39.0 °C or 38.0-38.9 °C with risk factors) is accompanied by signs such as maternal or fetal tachycardia, maternal leukocytosis, and purulent cervical discharge.

### 2.8 Statistical analysis

Statistical analyses were carried out using SPSS version 26.0 (IBM, Armonk, NY, USA), GraphPad Prism version 9.0 (GraphPad Software, San Diego, CA, USA). Normally distributed continuous data were expressed as mean ± SD and compared using the two independent samples *t*-test. Non-normally distributed continuous data were presented as median (IQR) and analyzed using the Mann–Whitney *U* test. Categorical variables were reported as frequencies (percentages) and compared using the chi-square test or Fisher’s exact test. For multiple comparisons in microbiome differential abundance analyses, false discovery rate (FDR) correction using the Benjamini–Hochberg (BH) method was applied. Differential abundance analysis was performed using Linear Discriminant Analysis Effect Size (LEfSe) as the primary exploratory tool, with an LDA score threshold > 2 and *P* < 0.05 considered significant. ALDEx2 (v1.28) was additionally applied with CLR transformation and Wilcoxon rank-sum test, followed by BH correction for multiple testing. Taxa with we.eBH < 0.05 and a substantial effect size (|diff.btw|> 1) were considered robustly differential. Spearman’s rank correlation was used to assess correlations among gut microbiota, SCFAs, and clinical indicators. Multiple linear regression analysis was applied to examine the effect of SCFAs levels on neonatal umbilical cord blood IL-6 levels, adjusting for potential confounders including GBS colonization status, mode of delivery, and intrapartum antibiotic use. A two-sided *P* value < 0.05 indicated statistical significance.

## 3 Results

### 3.1 Subject screening and basic features

Initially, 99 fecal specimens of pregnant women were collected during the study period. Twelve cases were excluded due to unqualified fecal samples. Finally, fecal specimens from 87 pregnant women were analyzed, resulting in 45 cases in the GBS-positive group and 42 cases in the GBS-negative group. The study flowchart is presented in **Figure 1**. The clinical characteristics of the 87 pregnant women between the two groups were shown in **Table 1**. There were no differences between the two groups except with regard to intrapartum antibiotic exposure (*P* < 0.001).

**Table 1:** Demographic and clinical characteristics of GBS-positive and GBS-negative mothers.

Characteristics	GBS-positive (n=45)	GBS-negative (n=42)	*OR/MD (95%CI)*	*P*
Maternal age, years	29.7 ± 4.7	29.8 ± 4.6	-0.406 (-2.396~1.584)	0.686
Residence-urban, n(%)	35 (77.8)	36 (85.7)	0.583 (0.191~1.777)	0.340
Local residents, n(%)	40 (88.9)	38 (90.5)	0.842 (0.210~3.373)	0.808
Ethnicity-Han, n(%)	43 (95.6)	40 (95.2)	1.075 (0.145~7.997)	0.944
BMI at delivery, $\bar{x}$ ± *s*, kg/m^2^	24.6 ± 2.5	24.8 ± 2.3	-0.239 (-1.263~0.785)	0.644
Primipara, n(%)	14 (31.1)	10 (23.8)	0.692 (0.268~1.789)	0.446
Abortion times, n (% ≥ 3 times)	5 (11.1)	3 (7.1)	1.625 (0.363~7.267)	0.788
Regular antenatal screening, n(%)	37 (82.2)	37 (88.1)	0.625 (0.187~2.089)	0.443
Intrapartum antibiotic use, n (%)	31 (68.9)	11 (26.2)	6.240 (2.453~15.872)	<0.001

GBS, group B *Streptococcus*; BMI, body mass index.

### 3.2 Comparison of pregnancy outcome and clinical characteristics of newborns between two groups

As shown in **Table 2**, the results of pregnancy outcome (e.g., cesarean section, intrapartum temperature ≥ 38°C, premature rupture of membranes, chorioamnionitis, meconium-stained amniotic fluid, fetal distress, postpartum hemorrhage) and clinical characteristics of newborns (e.g., male gender, GA < 37 weeks, birth weight, birth weight percentile-Fenton, small for gestation age, Apgar score ≤ 7 at 5 min, early-onset GBS disease) showed that there were no significant differences between GBS-positive group and GBS-negative group. Umbilical cord blood IL-6 levels of newborns were significantly higher in the GBS-positive group than in the GBS-negative group (*P* = 0.029).

**Table 2:** Pregnancy outcome and clinical characteristics of newborns of GBS-positive and GBS-negative group.

Variables	GBS-positive (n=45)	GBS-negative (n=42)	*OR/MD* (95%*CI*)	*P*
Pregnancy outcome				
Cesarean section, n (%)	20 (44.4)	16 (38.1)	1.300 (0.552~3.061)	0.548
Intrapartum temperature≥38°C, n (%)	9 (20.0)	5 (11.9)	1.850 (0.565~6.054)	0.305
PROM, n (%)	10 (22.2)	7 (16.7)	1.429 (0.488~4.179)	0.514
Chorioamnionitis, n (%)	8 (17.8)	2 (4.8)	4.324 (0.862~21.692)	0.091
MSAF, n (%)	6 (13.3)	4 (9.5)	1.462 (0.382~5.591)	0.415
Fetal distress, n (%)	6 (13.3)	3 (9.5)	2.000 (0.467~8.571)	0.486
Postpartum hemorrhage, n (%)	2 (4.4)	1 (2.4)	1.907 (0.166~21.841)	0.526
*Ruminococcus* abundance, [*M* (*Q1,Q3)*], E-03	6.1 (0.6, 26.5)	14.9 (6.9, 45.3)	–	0.036
Butyrate concentration, $\bar{x}$ ± *s*, μg/mg	1.3 ± 0.5	1.7 ± 0.6	-0.417 (-0.640~-0.193)	<0.001
Clinical characteristics of newborns				
Male gender, n (%)	24 (53.3)	22(52.4)	0.963 (0.415~2.235)	0.929
GA < 37 wk, n (%)	9 (20.0)	7 (16.7)	1.250 (0.420~3.725)	0.688
Birth weight, [*M* (*Q1,Q3)*], gram	3025.0 (2390.0, 3555.0)	3225.0 (2777.5, 3567.5)	–	0.218
Birth weight percentile-Fenton, [*M* (*Q1,Q3)*], %	48.5 (13.8, 68.5)	56.9 (29.4, 67.6)	–	0.234
SGA, n (%)	10 (22.2)	6 (14.3)	1.714 (0.563~5.222)	0.340
Apgar score ≤ 7 at 5 min, n (%)	1 (2.2)	0 (0.0)	0.512 (0.416~0.629)	0.517
GBS-EOD, n (%)	2 (4.4)	0 (0.0)	0.506 (0.410~0.642)	0.265
Umbilical cord blood IL-6, [*M* (*Q1,Q3)*], pg/ml	27.7 (11.1,71.5)	15.4 (11.1,31.3)	–	0.029

GBS, group B *Streptococcus*; PROM, premature rupture of membranes; MSAF, meconium-stained amniotic fluid; GA, gestational age; SGA, small for gestation age; GBS-EOD, early-onset GBS disease; IL-6, interleukin-6.

### 3.3 Comparison of α-, β-diversity between the GBS-positive and GBS-negative groups

Alpha and beta diversity analyses were performed to evaluate the differences in composition of gut microbiota. Severala-diversity indexes including Ace, Shannon, Simpson, PCoA plots based on weighted Unifrac distance and Bray Curtis distance (**Supplementary Figures 1**–**5**) indicated no significant difference in species richness and diversity between the two groups. The rarefaction curves of ASVs (Sobs) for all samples in the GBS-positive and GBS-negative groups approached saturation with increasing sequence number, confirming the reliability of the data and its suitability for downstream bioinformatics analyses (**Supplementary Figure 6**).

### 3.4 Alteration of taxa between the GBS-positive and GBS-negative groups

**Figure 2:**
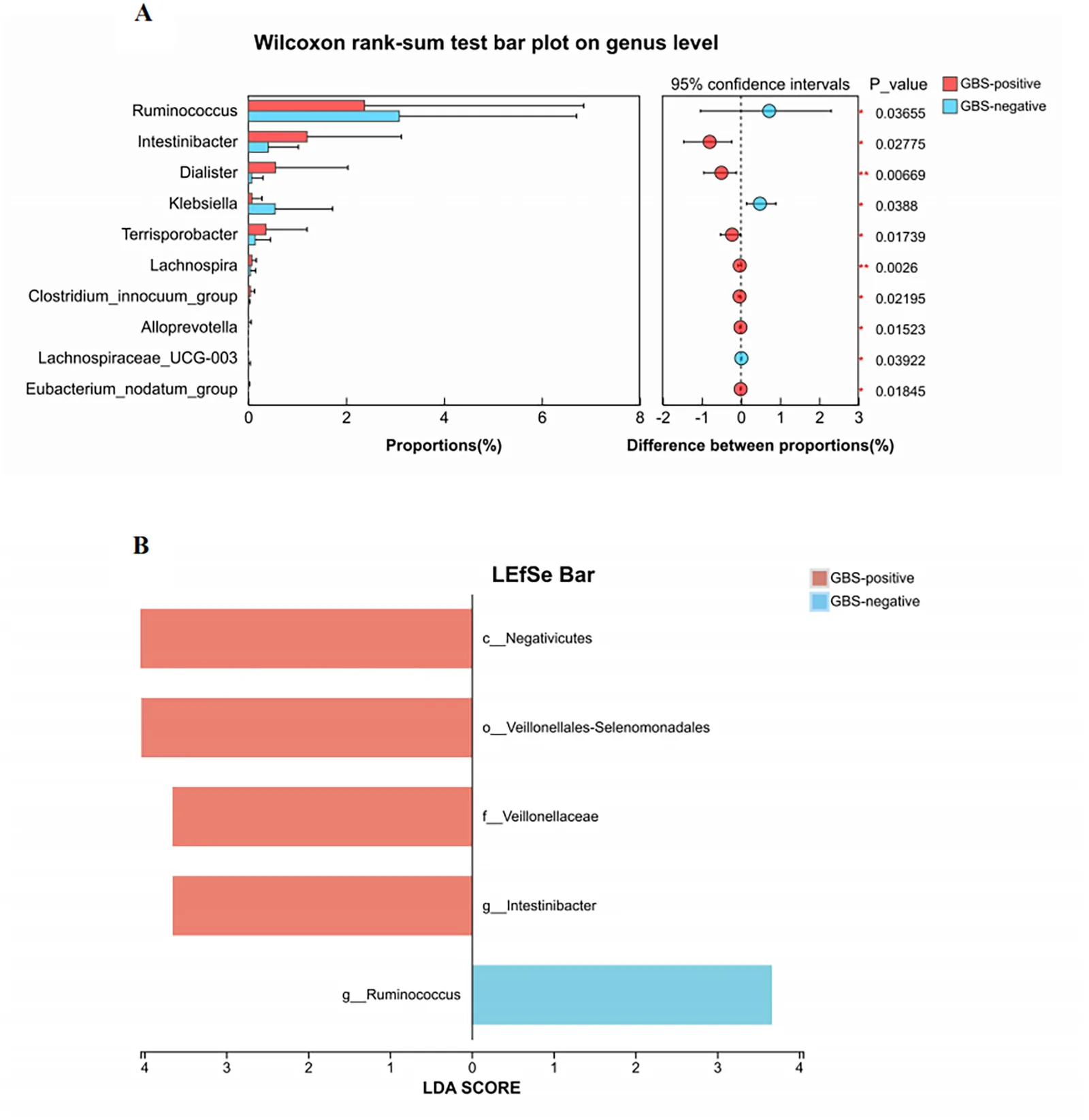
**(A)** Wilcoxon rank-sum test analysis at the genus level between the GBS-positive and GBS-negative groups. **(B)** LEfSe analysis (LDA>3.5) between the GBS-positive and GBS-negative groups from phylum level to genus level.

Wilcoxon rank-sum test identified ten genera with significantly different relative abundances between the two groups (all *P* < 0.05, **Figure 2A**). Among these, *Ruminococcus* and *Klebsiella* showed higher abundance in the GBS-negative group, whereas *Intestinibacter* and *Dialister* were enriched in the GBS-positive group (top two for each group). Ten genera showed nominal significance, which we present as suggestive trends and interpret with caution; however, after FDR correction, none of these taxa reached a *q*-value < 0.05 (**Supplementary Table 1**). Linear discriminant analysis (LDA) and Effect Size (LEfSe) was used to identify differentially abundant microbial taxa between the two groups. A total of five differentially abundant taxa were identified with LDA score >3.5, including the class Negativicutes, the order Veillonellales-Selenomonadales, the family Veillonellaceae, and the genera *Intestinibacter* and *Ruminococcus* from Phylum level to Genus level (**Figure 2B**). Notably, the abundance of *Ruminococcus* was significantly reduced in the GBS-positive group compared with the GBS-negative group (LDA = 3.669, *P* = 0.036). Although LEfSe highlighted *Ruminococcus* as a taxon with the highest LDA score indicating discriminative potential, this trend was only observed at the nominal level in ALDEx2 (we.ep < 0.05; **Supplementary Table 2**) and did not survive multiple-testing correction (we.eBH > 0.05). Thus, *Ruminococcus* is interpreted as a candidate genus with a differential trend rather than a definitively established differential taxon.

### 3.5 Comparison of SCFAs between the GBS-positive and GBS-negative groups

**Figure 3:**
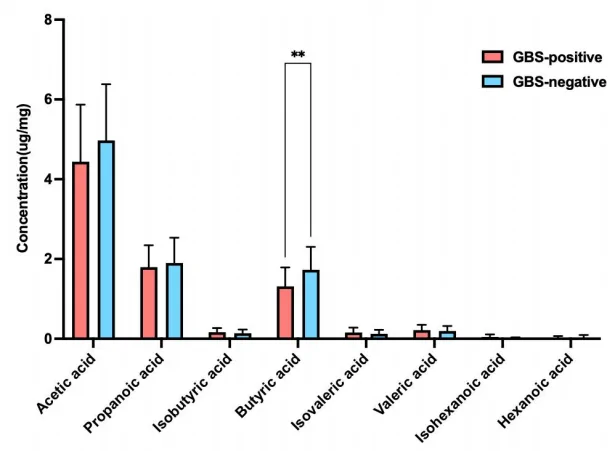
Comparison of SCFAs concentrations between the GBS-positive and GBS-negative groups. **P < 0.01.

The levels of eight SCFAs – acetic acid, propanoic acid, isobutyric acid, butyric acid, isovaleric acid, valeric acid, isohexanoic acid, and hexanoic acid-were compared between the GBS-positive and GBS-negative groups (**Figure 3**). The GBS-negative group showed slightly higher concentrations of acetic acid (*P* = 0.052) and significantly higher concentrations of butyric acid (*P* = 0.005) compared to the GBS-positive group, whereas the remaining five SCFAs were comparable between the two groups.

### 3.6 Spearman correlation analysis of clinical indicators, *Ruminococcus* abundance, and butyrate concentration

**Figure 4:**
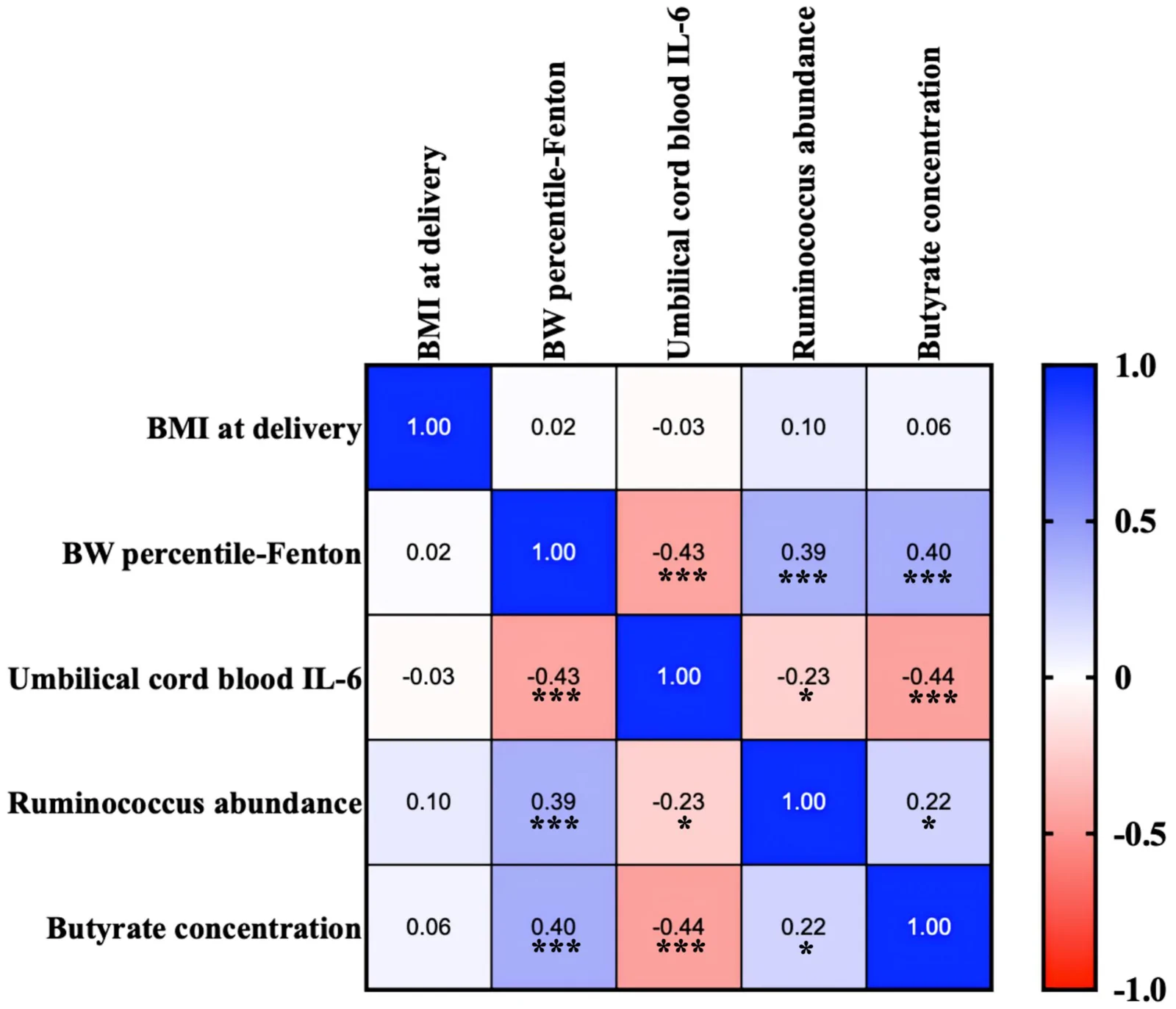
Variable correlation coefficient matrix heatmap. The Spearman correlation coefficient is calculated by assessing the relationship between each index (clinical indicators, *Ruminococcus* abundance, and butyrate concentration). The correlation effect is indicated by a color gradient from blue (negative correlation) to red (positive correlation). *P < 0.05, ***P < 0.001.

Given that *Ruminococcus* was the most differentially abundant genus and butyric acid was the most differential SCFAs between the two groups, we further performed Spearman correlation analyses to assess their relationships with clinical indicators associated with neonatal prognosis (**Figure 4**). A heatmap of the correlation matrix revealed that *Ruminococcus* abundance and butyrate concentration of pregnant women were positively correlated with birth weight percentile-Fenton of newborn (*ρ* = 0.390, *P* < 0.001; *ρ* = 0.403, *P* < 0.001) and negatively correlated with umbilical cord blood IL-6 level (*ρ* = -0.229, *P* = 0.033; *ρ* = -0.445, *P* < 0.001). *Ruminococcus* abundance were positively correlated with butyrate concentration among pregnant women (*ρ* = 0.216, *P* = 0.045).

### 3.7 Regression analysis of factors associated with neonatal umbilical cord blood IL-6

The multivariate analysis examined the associations between multiple factors and the outcome of neonatal umbilical cord blood IL-6 (**Table 3**). After adjusting for confounding factors including GBS colonization, intrapartum antibiotic use of pregnant women, cesarean section, birth weight percentile-Fenton, multivariate regression analysis showed that increased butyrate concentration served as an independent protective factor against elevated IL-6 (a*β*: -27.823, 95%*CI*: -50.041~-5.606), whereas the presence of chorioamnionitis was identified as an independent risk factor for elevated IL-6 (a*β*: 30.155, 95%*CI*: 2.556~57.754). However, increased *Ruminococcus* abundance did not emerge as an independent protective factor against elevated IL-6 (a*β*:3.203, 95% *CI*: -234.416 ~240.822).

**Table 3:** Multivariable linear regression analysis of perinatal factors associated with neonatal umbilical cord blood IL-6.

a*β* (95%*CI*)	*P* value	Multivariate analysis
-27.823 (-50.041~-5.606)	0.015	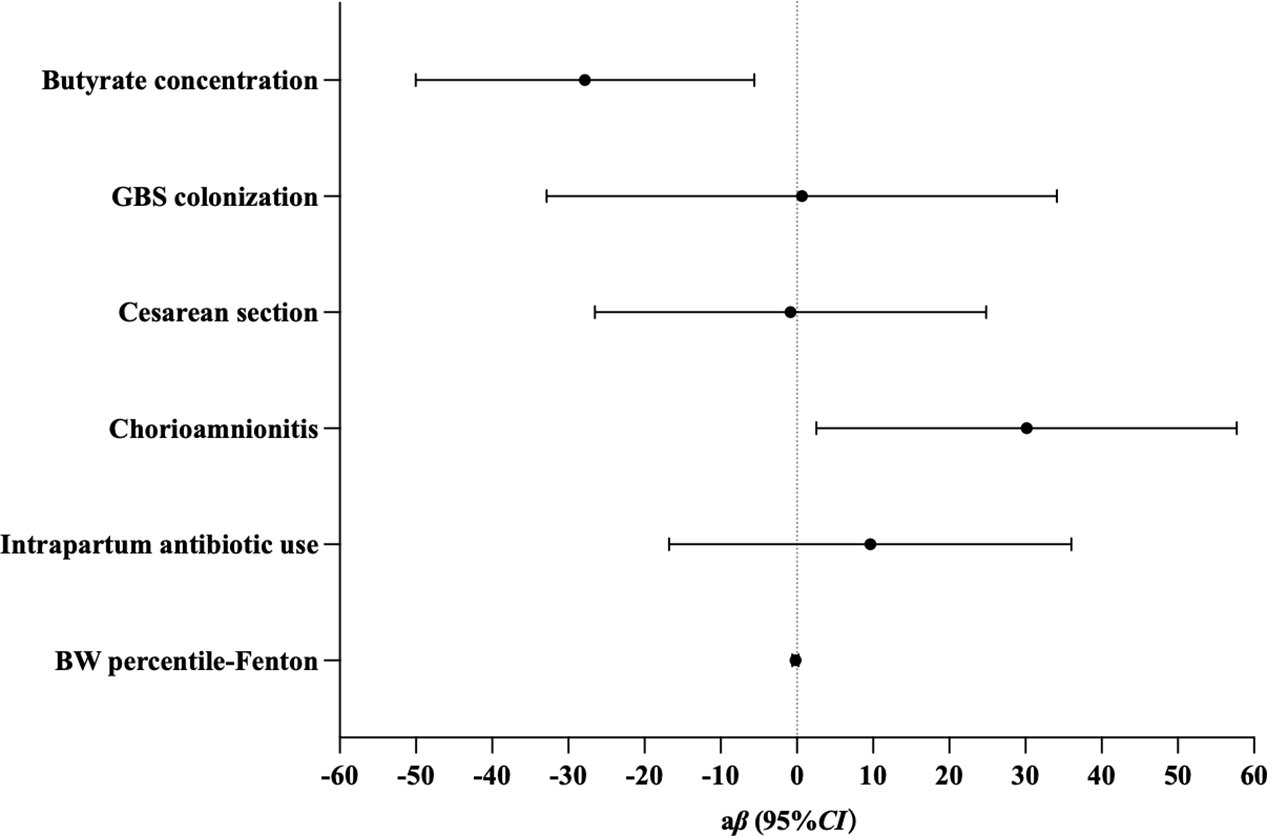
0.651 (-32.848~34.150)	0.969	
-0.834 (-26.507~24.838)	0.948	
30.155 (2.556~57.754)	0.033	
9.623 (-16.766~36.013)	0.466	
-0.198 (-0.623~0.228)	0.355	
Dependent variable:IL-6		

GBS, group B *Streptococcus*; BW, birth weight.

## 4 Discussion

This prospective cohort study systematically investigated alterations in gut microbiota composition and fecal SCFAs in women with late-pregnancy GBS colonization, and further analyzed their associations with neonatal inflammatory status and neonatal outcomes. The main findings were as follows: (1) GBS colonization was significantly associated with changes in the relative abundances of the genera *Ruminococcus*, *Intestinibacter*, and the class Negativicutes in the gut microbiota. (2) Fecal butyrate concentration was significantly lower in the GBS-positive group than in the GBS-negative group, and butyrate concentration was positively correlated with *Ruminococcus* abundance. (3) Both butyrate concentration and *Ruminococcus* abundance were positively correlated with neonatal birth weight and negatively correlated with umbilical cord blood IL-6 levels. (4) After adjusting for confounding factors including GBS colonization, regression analysis showed that increased butyrate concentration was an independent protective factor against elevated IL-6, whereas chorioamnionitis was an independent risk factor for elevated IL-6. These findings suggest a potential pathway connecting GBS colonization, gut dysbiosis, butyrate metabolism, and fetal inflammation. However, this hypothesis requires validation in future mechanistic studies, and clinical translation would necessitate rigorous interventional evaluation.

GBS is a globally important pathogen responsible for neonatal infections, yet it commonly and asymptomatically colonizes the gastrointestinal and lower genital tracts of women. GBS typically colonizes the gastrointestinal tract and vaginal mucosa; however, previous research has largely focused on its impact on the vaginal microbiota (Lim et al., 2021; Nadeau et al., 2022). The present study adopted an alternative perspective by exploring whether GBS colonization in late pregnancy influences the gut microbiota. Our findings revealed that GBS colonization is closely associated with alterations in the relative abundances of several genera. This observation is consistent with several recent studies. Wang et al. reported significant differences in the total number of species and microbial diversity between GBS-positive and healthy pregnant women, identifying multiple phyla (e.g., Lentisphaerae, Chlorobi, Fusobacteria) that were detected only in GBS-colonized individuals (Wang et al., 2022). Fang et al., studying a Chinese pregnant population, similarly confirmed that GBS colonization leads to gut microbiota disturbances, with PCoA revealing a distinct compositional difference between the GBS-positive and GBS-negative groups (Fang et al., 2023). Conversely, a study of a Japanese pregnant cohort reported that GBS colonization was associated with enrichment of Prevotella, Atopobium, and Gardnerella in the vaginal microbiota, but no significant changes in gut microbial diversity were observed (Song et al., 2025). These results suggest that the effect of GBS on the gut microbiota may exhibit inter-population heterogeneity, potentially attributable to differences in ethnicity, dietary habits, environmental factors, and the virulence gene profiles of GBS strains (Crestani et al., 2024; Ventura et al., 2026). Notably, the present study focused on *Ruminococcus*, a genus that was significantly altered in the GBS-positive group and is recognized as one of the important butyrate-producing bacteria in the gut. This finding provides a microbiological basis for subsequent analyses of aberrant butyrate metabolism. However, complementary ALDEx2 analysis revealed that this signal was marginal after multiple-testing correction (we.ep = 0.0553, we.eBH = 0.8100), despite a considerable effect size (diff.btw = -1.6335). This discrepancy likely reflects the limited statistical power inherent to our moderate sample size and the sparsity of microbiome data, wherein the conservative BH correction may over-penalize true but modest effects. Therefore, while our LEfSe findings offer exploratory evidence implicating *Ruminococcus*, they warrant cautious interpretation. Future large-scale longitudinal cohort studies with enhanced statistical power are essential to validate its potential role in the observed metabolic alterations.

SCFAs are the principal metabolites generated by gut microbial fermentation of dietary fiber. Among them, butyric acid has attracted considerable attention owing to its potent anti-inflammatory and immunomodulatory properties. Butyric acid exerts broad anti-inflammatory effects by activating G protein-coupled receptors, such as GPR41 and GPR43 (Ma et al., 2023; Yang et al., 2022), and by inhibiting histone deacetylase (HDACs) activity (Li et al., 2021). In the unique immune milieu of pregnancy, the maintenance of maternal intestinal SCFAs including butyrate levels is essential for establishing an anti-inflammatory microenvironment and preserving maternal-fetal immune tolerance (Uchida et al., 2025; Yao et al., 2025; Koren et al., 2024). Prior evidence indicates that the gut microbiota undergoes remodeling during normal pregnancy, characterized by an expansion of lactate-producing bacteria and a concomitant decline in butyrate-producing populations (Di et al., 2020; Ly et al., 2026). Specifically, late pregnancy exhibits increased lactic acid-producing bacteria alongside reduced Firmicutes-to-Bacteroidetes ratios (Ly et al., 2026), with notable decreases in representative butyrate-producing taxa such as Lachnospiraceae and Ruminococcaceae (Uchida et al., 2025). Although the physiological significance of this shift remains to be fully elucidated, it may render intestinal immune homeostasis particularly susceptible to perturbation during gestation. In the present study, fecal butyrate concentrations were significantly lower in GBS-positive pregnant women than in GBS-negative controls and positively correlated with the abundance of the genus *Ruminococcus*, which includes known butyrate-producing members such as *R. bromii.* These findings suggest a potential association between GBS colonization, lower *Ruminococcus* abundance, and reduced butyrate levels. Whether this association is mediated by disruption of the normal gut microbiota succession during pregnancy warrants further investigation. However, owing to the genus-level resolution of 16S rRNA sequencing, the specific species driving this association remain undefined.

The present study also found that both butyrate concentration and *Ruminococcus* abundance were significantly positively correlated with neonatal birth weight. This finding aligns with the recognized role of SCFAs in fetal growth regulation. First, as primary metabolites of the gut microbiota, SCFAs are absorbed by intestinal epithelial cells and then enter the systemic circulation for subsequent transport to the fetus across the placenta (Qin et al., 2025). Second, fluctuations in maternal SCFA levels can influence fetal metabolic reprogramming, with SCFA dysregulation under metabolic stress conditions such as diabetes and obesity being capable of adversely affecting fetal cardiovascular, neurological, and immune development (Feng et al., 2025). Recent studies have further demonstrated a significant association between infant fecal SCFA levels and neonatal birth weight, with higher SCFA levels in 4-week-old infants correlating with greater birth weight (Szczuko et al., 2025). Moreover, in GBS-exposed infants, alterations in the gut microbiota and metabolites mediate the effect of maternal GBS on infant length growth, leading to lower length-for-age z-scores at two months of age (Li et al., 2025). Complementing these findings, our study moves the observation window to the prenatal period and reveals a positive association between maternal gut butyrate levels and neonatal birth weight, suggesting that maintaining adequate butyrate levels during late pregnancy may help optimize fetal growth.

The present study further revealed that butyrate concentration was significantly negatively correlated with neonatal umbilical cord blood IL-6 level and identified elevated butyrate concentration as an independent protective factor against increased IL-6. These findings are robustly supported by evidence from animal studies. Yong et al. demonstrated in a pre-eclampsia rat model that exogenous sodium butyrate supplementation significantly reduced maternal serum levels of IL-1β, IL-6, and TGF-β, while simultaneously increasing placental weight and improving intestinal barrier function (Yong et al., 2022). More importantly, Uchida et al. confirmed in a dysbiosis-induced preterm birth mouse model that butyrate supplementation reduced the incidence of preterm birth, prolonged gestational duration, and restored Treg numbers (Uchida et al., 2025). In the human cohort of that study, preterm birth cases exhibited a marked reduction in the combined abundance of the representative butyrate-producing families Lachnospiraceae and Ruminococcaceae; this combined abundance was independently associated with the risk of spontaneous preterm birth and positively correlated with gestational age. Collectively, these data from different models and populations consistently indicate that butyrate deficiency is closely linked to aggravated inflammatory status during pregnancy and adverse perinatal outcomes, whereas butyrate supplementation exerts a significant protective effect.

Umbilical cord blood IL-6 has been extensively validated as an important biomarker of fetal inflammatory response syndrome (FIRS) and perinatal inflammation. A recent narrative review concluded that elevated umbilical cord blood IL-6 levels are closely associated with early-onset neonatal sepsis (EONS) and respiratory complications (Vasilescu et al., 2025). Chaiworapongsa et al. reported that the median umbilical venous blood IL-6 concentration in newborns born to women with clinical chorioamnionitis was as high as 27.46 pg/mL, compared with only 2.13 pg/mL in controls (Chaiworapongsa et al., 2002). In the present study, chorioamnionitis was identified as an independent risk factor for elevated IL-6, which is highly consistent with the aforementioned findings. Although chorioamnionitis lies on the causal pathway between GBS colonization and elevated cord blood IL-6, we adjusted for it in our regression models to estimate the independent effect of GBS colonization beyond its well-recognized complication. Nonetheless, we acknowledge that this adjustment may partially block the biological pathway and should be interpreted with caution. Future studies employing formal mediation analyses are warranted to disentangle the direct and indirect effects of GBS colonization, chorioamnionitis, and the gut microbiota-SCFA axis on neonatal inflammatory status.

This study presents several innovations. It is a prospective investigation to link the gut microbiota-SCFAs axis in GBS-colonized pregnant women to neonatal inflammatory outcomes, and it identifies butyrate as an independent protective factor by integrating multi-omics and clinical data with multivariate regression. This study propose a hypothesis-generating model linking GBS colonization, reduced *Ruminococcus* abundance, decreased butyrate, and elevated IL-6. However, several limitations must be acknowledged. (1) A limitation of this study is the relatively modest sample size, which may reduce statistical power for detecting differences in low-abundance taxa. Consequently, our findings should be interpreted as exploratory and hypothesis-generating, warranting validation in larger multicenter cohorts and mechanistic investigation in animal models. (2) We excluded women with obesity, diabetes, and hypertensive disorders to minimize confounding from metabolic conditions known to affect gut microbiota and SCFAs metabolism, prioritizing internal validity in this exploratory assessment of GBS-specific associations. However, this selection limits generalizability, particularly given that obesity and diabetes are risk factors for GBS colonization. Our findings therefore reflect a low-risk pregnancy context and warrant validation in more diverse populations. (3) Although 89.7% of participants were local residents of Xiamen city, and the two groups were comparable in terms of geographic origin, fecal SCFA levels are substantially influenced by dietary fiber intake and overall dietary patterns. Therefore, the absence of detailed information on maternal dietary habits during pregnancy remains an important limitation. (4) Although intrapartum antibiotic use was adjusted for, its types and durations were not further stratified. (5) The genus *Ruminococcus* includes functionally distinct species, such as butyrogenic *R. bromii* and mucinolytic *R. gnavus*. Due to the inherent resolution and compositional limitations of 16S rRNA sequencing, we could neither identify the specific species driving the observed correlations nor determine whether the lower *Ruminococcus* relative abundance represents an absolute decrease or reflects broader community shifts. Future shotgun metagenomic or full-length 16S sequencing studies are needed to clarify species-level functional roles and absolute abundance changes. (6) The cross-sectional single fecal sampling cannot capture dynamic microbial changes, and the lack of vaginal microbiota data limits assessment of gut-vagina interactions. Moreover, the observational design precludes determining whether reduced *Ruminococcus*/butyrate directly increases IL-6 or is a downstream effect of GBS-associated dysbiosis. Future mechanistic studies (e.g., gnotobiotic models, *in vitro* butyrate stimulation) are required to establish causation. (7) Given the conservative nature of FDR correction and the sparsity of microbiome data, the nominal trends observed for several taxa that did not survive adjustment are hypothesis-generating in nature and warrant further validation through larger external cohorts rather than being interpreted as confirmatory findings.

## 5 Conclusion

In summary, this study reveals a significant association between late-pregnancy GBS colonization, gut dysbiosis, and aberrant butyrate metabolism. Lower intestinal butyrate concentration is independently associated with elevated umbilical cord blood IL-6 levels, a key marker of aggravated neonatal inflammation. Our findings highlight a plausible association between GBS-related gut microbial alterations, decreased butyrate, and elevated cord blood IL-6. Whether butyrate serves as a causal link in this pathway requires confirmation in mechanistic and interventional studies. These observations provide insights into host–microbe interactions in GBS colonization and a rationale for future mechanistic studies, but clinical translation would require rigorous interventional validation.

## Data Availability

The datasets presented in this study can be found in online repositories. The names of the repository/repositories and accession number(s) can be found in the article/**Supplementary Material**.
