# Supplementary material for: Gut microbiota and short-chain fatty acids in pregnant women with group B Streptococcus colonization and their impact on neonatal outcomes
Source: Front Cell Infect Microbiol. 2026 Sep 10;16:1912234. doi: 10.3389/fcimb.2026.1912234 (PMC13600864; doi:10.3389/fcimb.2026.1912234)
Supplement: Supplementary file 1 [file Table1.docx]

**Supplementary Table S1 Nominally significant genera and FDR *q*-values**

| Factor | GBS-positive  mean ± SD (%) | GBS-negativemean ± SD  (%) | *P*_value | *P*_adjust | Effect size |
| --- | --- | --- | --- | --- | --- |
| g__Ruminococcus | 2.38 ± 4.48 | 3.09 ± 3.62 | 0.03655 | 0.7161 | -0.7228 |
| g__Intestinibacter | 1.21 ± 1.93 | 0.41 ± 0.62 | 0.02775 | 0.7161 | 0.7979 |
| g__Dialister | 0.56 ± 1.48 | 0.07 ± 0.23 | 0.00669 | 0.7161 | 0.4987 |
| g__Klebsiella | 0.08 ± 0.21 | 0.55 ± 0.18 | 0.0388 | 0.7161 | -0.4848 |
| g__Terrisporobacter | 0.37 ± 0.84 | 0.14 ± 0.31 | 0.01739 | 0.7161 | 0.2257 |
| g__Lachnospira | 0.08 ± 0.09 | 0.05 ± 0.11 | 0.0026 | 0.7161 | 0.03064 |
| g__Clostridium_innocuum_group | 0.05 ± 0.08 | 0.01 ± 0.03 | 0.02195 | 0.7161 | 0.03514 |
| g__Alloprevotella | 0.01 ± 0.05 | 0.00 ± 0.00 | 0.01523 | 0.7161 | 0.01318 |
| g__Lachnospiraceae_UCG-003 | 0.00 ± 0.00 | 0.01 ± 0.04 | 0.03922 | 0.7161 | -0.00986 |
| g__Eubacterium_nodatum_group | 0.01 ± 0.02 | 0.00 ± 0.00 | 0.01845 | 0.7161 | 0.00817 |

**Supplementary Table S2 ALDEx2 significant difference results at the Genus level**

| Factor | we.ep | we.eBH | diff.btw |
| --- | --- | --- | --- |
| g__Agathobacter | 0.0357078923661321 | 0.781898408724563 | -1.32567116158762 |
| g__Dialister | 0.0418392734615679 | 0.727601823494347 | 2.33116469420396 |
| g__Eubacterium_hallii_group | 0.0481792328865077 | 0.812853647585918 | -0.343597654984919 |
| g__Lachnospira | 0.0164558525733112 | 0.638656835911428 | 2.50531848252756 |
| g__Ruminococcus | 0.0453177241554526 | 0.730039303721052 | -1.80349493138127 |
| g__Terrisporobacter | 0.0484911213212291 | 0.765247602251364 | 2.27133442844046 |
| g__unclassified_f__Lachnospiraceae | 0.0213332785789334 | 0.750761498073902 | 0.512273744020229 |
